# Don’t die like me: Which proteins are responsible for the selective neuronal vulnerability within the *substantia nigra*?

**DOI:** 10.1371/journal.pone.0296730

**Published:** 2024-07-31

**Authors:** Simone Steinbach, Mariana Molina, Lea T. Grinberg, Luisa Aring, Annika Guntermann, Katrin Marcus, Helmut Heinsen, Renata E. Paraizo Leite, Caroline May

**Affiliations:** 1 Medizinisches Proteom-Center, Center of Protein Diagnostics (ProDi), Ruhr-Universität Bochum, Bochum, Germany; 2 Physiopathology in Aging Lab/Brazilian Aging Brain Study Group-LIM22, University of São Paulo Medical School, São Paulo, Brazil; 3 Discipline of Pathophysiology, University of São Paulo Medical School, São Paulo, Brazil; 4 Department of Neurology, Memory and Aging Center, University of California, San Francisco, San Francisco, California, United States of America; 5 Institute of Forensic Pathology, Würzburg, Germany; 6 Discipline of Geriatrics, University of São Paulo Medical School, São Paulo, Brazil; University Hospital Wurzburg, GERMANY

## Abstract

A hallmark of Parkinson’s disease is the specific degeneration of dopaminergic neurons in the *substantia nigra pars compacta*. Interestingly, not all of these neurons are affected to the same extent. Studies revealed that neurons located more ventrally within the *substantia nigra pars compacta* have a higher prevalence to degenerate than those located in the dorsal tier. The underlying reasons for this selective neuronal vulnerability are still unknown. The aim of the present study was to gain a better understanding of molecular differences between these two neuronal subpopulations that may explain the selective neuronal vulnerability within the human *substantia nigra*. For this purpose, the neurons from the ventral as well as dorsal tier of the *substantia nigra* were specifically isolated out of neuropathologically unremarkable human *substantia nigra* sections with laser microdissection. Following, their proteome was analyzed by data independent acquisition mass spectrometry. The samples were analysed donor-specifically and not pooled for this purpose. A total of 5,391 proteins were identified in the *substantia nigra*. Of these, 2,453 proteins could be quantified in 100% of the dorsal tier samples. 1,629 could be quantified in 100% of the ventral tier samples. Nine proteins were differentially regulated with a log2 value ≥0.5 and a Qvalue ≤0.05. Of these 7 were higher abundant in the dorsal tier and 2 higher in the ventral tier. These proteins are associated with the cytoskeleton, neuronal plasticity, or calcium homeostasis. With these findings a deeper understanding can be gained of the selective neuronal vulnerability within the *substantia nigra* and of protective mechanisms against neurodegeneration in specific neuronal subpopulations.

## Introduction

In recent years, Parkinson’s disease (PD) has become a major topic in research due to the increasing number of patients caused by the increasing human life expectancy [1]. In PD dopaminergic neurons within the *substantia nigra* are primarily affected [2]. This so called selective neuronal vulnerability (SNV) can also be observed in Alzheimer’s or Huntington’s disease, where other neuronal populations are primarily affected at the onset of the disease. However, reasons for the SNV in PD as well as in other neurodegenerative diseases are still an open question. It is assumed that morphological characteristics of these neuronal populations, such as a large cell surface and long axons that project over long distances to other brain regions, may contribute to their higher susceptibility [3]. Dopaminergic neurons within the *substantia nigra* are characterized by long axons (~47 cm) that project to the striatum [4, 5]. The vulnerability of large neurons with long axons may be enhanced by their high energy requirement to maintain their functionality, such as axonal transportation [6]. Furthermore, disruptive mechanisms to their cytoskeleton may lead to increased susceptibility, e.g., by aggregation of axonal neurofilaments or microtubule-associated proteins such as tau [3]. However, it is not only morphological features that can influence the SNV of neurons. The neuronal circuits in which neurons are involved, and thus their molecular properties, can also influence their SNV [7]. For example, dysregulated synaptic molecular pathways could affect not only a neuron but also its neighboring neurons [2]. Other molecular features that distinguish neuronal subpopulations with different SNVs may be their neurotransmitter [3]. For example, the neurotransmitter dopamine is expressed in neurons that are particularly affected in PD. Dopamine appears to induce oxidative stress in presynaptic terminals, one of the most vulnerable regions of neurons [2]. Such findings highlight the need to study neurons in the complex network they form with each other and with additional cell types (such as microglia, oligodendrocytes or astrocytes) [3]. This may lead to a better understanding of SNV and thus of neurodegenerative diseases such as PD.

The complexity of the molecular mechanisms underlying SNV in PD is even more specific. In PD, neurodegenerative processes do not affect all dopaminergic neurons within the substantia nigra equally. Neurons in the ventral layer of the substantia nigra are more susceptible to neurodegeneration in PD than those in the dorsal layer of the *substantia nigra* [8–10]. According to Fearnley et al., the loss of pigmented neurons in the ventral layer of the substantia nigra is up to 91%, while it is only about 56% in the dorsal layer of the substantia nigra during PD progression [9]. Similar observations were made by Gibb *et al*. [11]. They found a complete loss of ventrolateral neurons in the *substantia nigra* and a moderate loss of neurons within the dorsal *substantia nigra*. Although it has been known for several decades, the reasons for this SNV are still not fully understood. It has been hypothesized that the expression of specific proteins, such as calbindin D28K, may have a neuroprotective effect on neurons located in the dorsal layer of the *substantia nigra* [12]. However, a proteomic investigation of these single neuronal populations was not possible in the past due to a lack of possible isolation methods. This requires an isolation technique that allows accurate and precise enrichment of single neurons from the heterogeneous brain tissue, which has not been available in the past.

Traditional enrichment and isolation techniques were not suitable for this purpose. These techniques involved either tissue homogenization, which results in a mixture of all cells present in the tissue, or chemical and mechanical processing [13]. Nowadays microdissection and especially laser microdissection (LMD) allows precise and contamination free isolation of cell clusters or single cells out of a heterogeneous tissue [14]. For example, Kamath et al. enriched dopamine neurons from patients with PD and matched controls, profiled them transcriptionally by single-nucleus RNA-sequencing. By this, they could identify neuronal subtypes based on gene expression [15]. For the present study, LMD was the method of choice [16]. It allows a microscopic isolation and enrichment of specific neuronal populations of the *substantia nigra*. In LMD a highly focused UV laser beam cuts out cells of interest that have been previously marked by microscopic visualization. During the UV cutting, a thin line of tissue is removed around each cell of interest. This separates the cells of interest from the surrounding material and then collects them in a reaction tube [14, 16, 17]. Based on this approach, we specifically enriched neuronal cell bodies located in the dorsal tier and neuronal cell bodies located in the ventral tier of the *substantia nigra*. Subsequently, the proteomes of the enriched neuronal cell bodies were analyzed by data independent acquisition (DIA) mass spectrometry and the proteomes of both neuronal subpopulations were compared.

Interestingly, proteins were identified that were either specific or higher expressed in one of the examined neuronal subpopulations within the *substantia nigra*. While differentially expressed proteins of neurons located in the dorsal tier are mainly involved in the maintenance of the cytoskeleton; specific or differentially expressed proteins of the neurons located in the ventral tier are associated with the calcium homeostasis and the aggregation of alpha-synuclein. These findings lead to a new hypothesis, which indicates that the molecular processes maintaining the stability of the cytoskeleton may be involved in the SNV of neuronal populations in the *substantia nigra*.

## Material and methods

**Fig 1:**
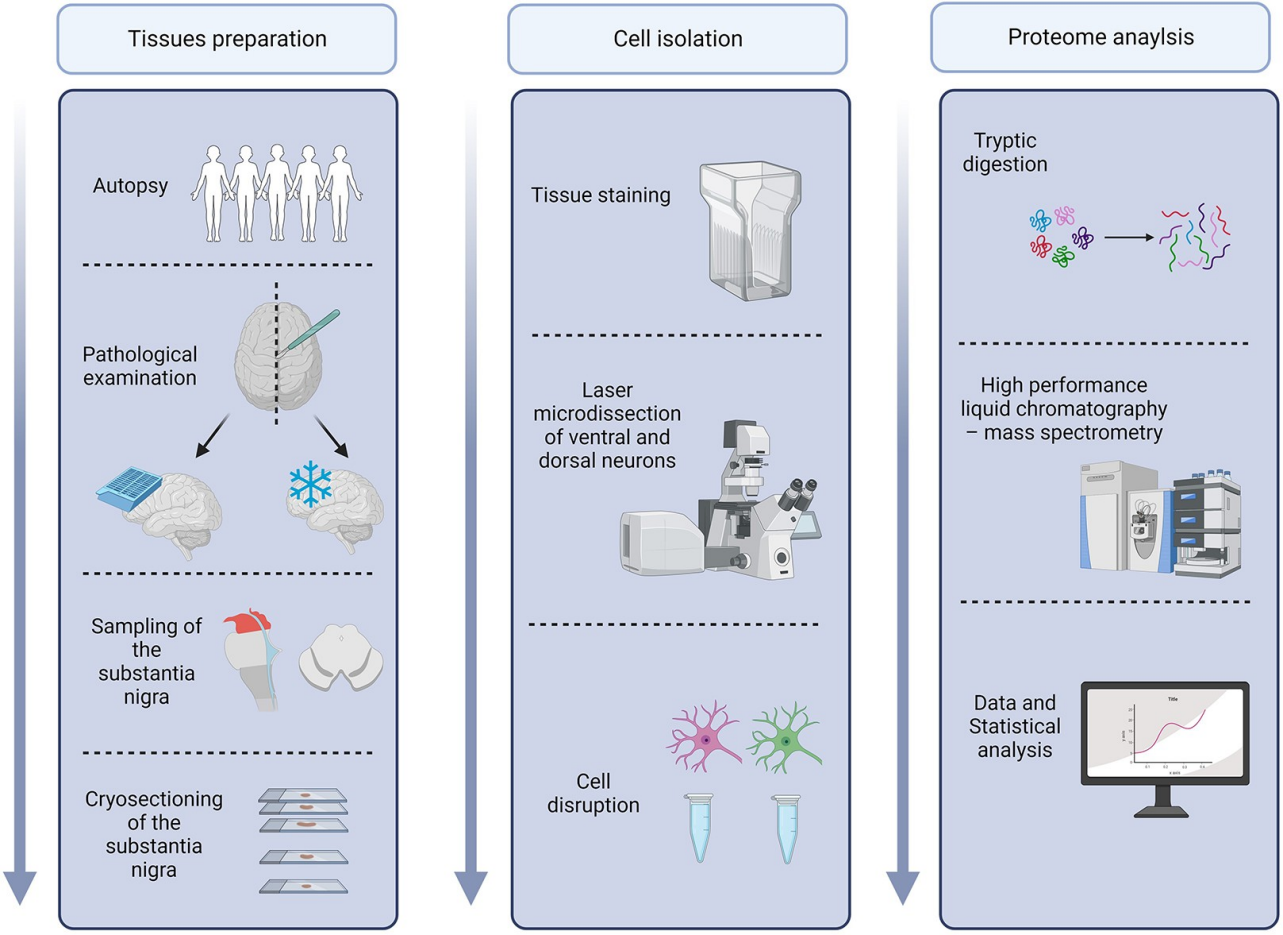
Schematic overview of the study workflow. Created with BioRender.com.

To investigate the selective neuronal vulnerability of the *substantia nigra*, a LMD-based approach was combined with DIA mass spectrometry and subsequent statistical data analysis. The study design is shown in Fig 1.

Analyses were cell specific (dorsal and ventral neurons) and donor specific.

### Subjects

Five post mortem human *substantia nigra* tissues were provided by the Biobank for Aging Studies at the University of São Paulo Medical School. Brain tissue donors were neurologically and histopathologically healthy individuals aged ≥ 50 years who died of natural causes (Table 1). The ethics committees of the Ruhr University Bochum (4760–13) and the University of São Paulo (CONEP B-025) approved the use of human postmortem brain tissue. A written informed consent was signed by the next of kin for each postmortem sample.

**Table 1:** Subjects.

Gender (m/f)	Age (years)	PMI (hours)
M	57	19.2
F	80	18.2
F	76	16.0
M	70	13.4
M	84	15.9

Abbreviations: f = female, m = male, PMI = post mortem interval

The demographic data of the donors were recorded by the Biobank for Aging Studies of the Faculty of Medicine of the University of São Paulo. Sociodemographic (smoking, status, education, age, etc.) and disease-related facts were documented by interviewing relatives using a standardized protocol. Neuropathology was performed by a consultant neuropathologist.

### Sampling

*Substantia nigra* tissues were collected according to the protocol of the Biobank for Aging Studies at the University of São Paulo Medical School [18]. After sampling, *substantia nigra* tissues were immediately snap frozen in liquid nitrogen and stored at -80°C.

### Cryosectioning

The *substantia nigra* tissues were sectioned using a Cyrostat Microm HM550 (Thermo Scientific, Dreieich, Germany) with a fixed knife holder (Leica Biosystems, Nußloch, Germany). The object temperature of the cryostat was set to -10°C and the chamber temperature to -20°C. Prior to sectioning, tissue was left in the cryostat for warming up to -20°C. Then, 10 μm thick sagittal orientated sections were produced, placed on membrane slides for LMD (1.0 PEN membrane slides, Carl Zeiss Microscopy GmbH, Göttingen, Germany) and stored at -80°C.

### Staining

Sections were stained with cresyl violet according to the protocol of Carl Zeiss Microscopy GmbH with slight modifications. In short, sections were incubated for 2 min in 4°C cooled 70% (v/v) ethanol (Merck KGaA, Darmstadt, Germany). After this, they were stained with 500 μl 1% (w/v) cresyl violet solution (1 g of cresyl violet acetate (Sigma Life Sciences, St. Louis, USA) dissolved in 50% (v/v) ethanol) for 20 sec. The cresyl violet solution was then discarded and the sections were washed by dipping them three times in 4°C cooled 70% (v/v) ethanol and one time in 4°C cooled 100% (v/v) ethanol. Finally, sections were air dried for 2 min.

### Laser microdissection

Laser microdissection was performed directly after staining. Slides were placed on the PALM Micro Beam instrument (P.A.L.M.-System LCM, Carl Zeiss Microscopy GmbH). Neurons of the *substantia nigra* were marked at a magnification of 400 using the software PALMRobo 4.6 pro (Carl Zeiss Microscopy GmbH, Göttingen, Germany). Cutting and catapulting of neurons were carried out by the option “RoboLPC”. Energy settings lay between 34–40 for cutting and 23–27 for LPC, depending on the room climate. Neurons were catapulted into the cap of a non-adhesive reaction tube (MicroTube 500, Carl Zeiss Microscopy GmbH, Göttingen, Germany) that was filled with 48 μl ultrapure water (TKA-Gene PURE, Thermo Scientific, Baltimore, USA). For each case ~1,500,000 μm^2^ neuronal tissue was collected. For this, between three and ten sections of each case were needed. After sampling, reaction tubes were stored upside down at -80°C.

### Tryptic digestion

Tryptic digestion was performed according to the protocol of Molina *et al*. with some changes [16]. In short, directly before the digestion was performed frozen samples were centrifuged for 1 min at 2,655 G and 4°C (Centrifuge 5415R, Eppendorf GmbH, Hamburg, Germany). Then, 7 μl of 1% RapidGest^TM^ SF Surfactant (Waters GmbH, Milford, MA, USA; resuspended in 50 mM ammonium bicarbonate (Sigma-Aldrich, Munich, Germany)) were added to the samples. Incubation was performed for 30 min at room temperature and for another 30 min at 60°C. Next, samples were incubated with 1 μl of 250 mM 1,4-dithiothreitol (AppliChem GmbH, Darmstadt, Germany) for 30 min at 60°C. After samples were cooled down to room temperature (15 min), they were incubated in the dark with 1.4 μl of 0.55 M iodoacetamide (AppliChem GmbH, Darmstadt, Germany) for 30 min. Next, samples were incubated at 95°C for 5 min and cooled down again to room temperature for 15 min. Tryptic digestion was initiated by adding 7 μl trypsin solution (0.012 μg/μl trypsin in 50 mM ammonium bicarbonate and 3 mM acetic acid; Promega, Mannheim, Germany) to the samples. Digestion was performed for 4 h at 37°C and stopped with 3.25 μl 10% trifluoroacetic acid (TFA, Sigma Aldrich GmbH, Hofheim, Germany). After 30 min at 37°C samples were centrifuged for 15 min at 4°C and 20,817 G. Supernatants were transferred into glass vials for mass spectrometric (MS) analysis (CS Chromatographie Service GmbH, Langerwehe, Germany) and dried in a vacuum concentrator (Concentrator plus, Eppendorf GmbH, Hamburg, Germany). Finally, samples were filled up with 0.1% TFA to a volume of 35 μl. Following, 2 μl of each sample were used to determine the peptide concentration using an amino acid analysis. For the following MS analysis 800 ng peptides were diluted in 15 μl of 0.1% (v/v) TFA.

### High performance liquid chromatography–mass spectrometry

In this study, a DIA mass spectrometric approach was performed and the resulting data were analyzed using the software Spectronaut^®^ Pulsar (Biognosys AG, Schlieren, Switzerland). Therefore, 1 injection volume of iRT peptides (internal retention time peptides, Biognosys AG, Schlieren, Switzerland) was added to each of the five individual samples of dorsal neurons and ventral neurons.

High performance liquid chromatography (HPLC) analysis was performed on an UltiMate 3000 RSLC nano system (Dionex, Idstein, Germany). Samples were injected into the system using an autosampler, with 0.1% TFA at 60°C and a flow rate of 30 μl/min. Peptides were pre-concentrated on a trap column (PepMap100, C18 phase, 100 μm ID x 2 cm, particle size 5 μm, pore size 100 Ǻ) for 7 min and then transferred on to the analytical column. Peptides were separated on the analytical column (PepMap C18, C18 phase, 75 μm ID x 50 cm, particle size 2 μm, pore size 100 Å) by an 141 min segmented gradient from 5%-60% (v/v) running buffer B (detailed gradient information can be found in Table 2). The solvent system was as following: running buffer A: 0.1% (v/v) formic acid, and running buffer B: 84% (v/v) acetonitrile, 0.1% (v/v) formic acid. The HPLC system was online connected to a Q Exactive HF (Thermo Fisher Scientific, Bremen, Germany). An electrospray ionization was performed to inject the eluting peptides into the mass spectrometer. For this, the ion spray voltage was set to 1,600 V (+) and the capillary temperature to 250°C.

**Table 2:** HPLC gradient.

Time (min)	Running buffer B (%)
0	5
7	5
20	8
30	10
45	12
75	15
105	19
113	21
118	23
121	25
124	27
127	30
129	34
132	37
136	43
142	52
148	60
149	95
154	95
155	5
160	5

Mass spectrometry was performed in DIA mode. The scan range for full scans was set to 350–1,100 m/z. For MS/MS scans this scan range was divided into 40 isolation windows, each with a wide of 20 m/z and an overlap of 1 m/z. Full scans were performed with a resolution of 120,000, an AGC target of 3e6 and a maximum injection time of 20 ms. Fragment ions were generated by HCD with an NCE of 25.5%, 27% and 30%. MS/MS scans were generated with a resolution of 30,000, an AGC of 3e6, and an injection time of ‘auto max’. The default charge state was set to ≥ +2. Further, the lock mass of polydimethylcyclosiloxane (m/z: 445.12) was defined and the fixed first mass was set to 200 m/z.

Quality control of the mass spectrometric measurements was ensured by two approaches. First, in regular intervals MS standard samples were measured; second, due to the spiked iRT labeling of the samples peptides. By this iRT labeling identified peptides were calibrated within the software Spectronaut^®^ Pulsar regarding intensity, retention time adjustment as well as m/z calibration.

### Data analysis and statistics

The generated DIA data of ten individual samples were analyzed via the software Spectronaut^®^ Pulsar according to the manufactures default settings with some modifications. The retention time prediction was performed using ‘dynamic iRT’ and a correction factor of 1. Local mass calibration was used and decoys were generated by the scramble option. The fragment quantification on the MS/MS level was performed by interference correction with a minimum of three fragment ions. False discovery rate (FDR) was calculated using the algorithm mProphet and its cutoff was set to 1% on the peptide level. Protein inference was achieved by applying the ID picker algorithm. As reference spectral library an inhouse generated spectral library for human *substantia nigra* was applied. Enzyme specificity was set to trypsin and two missed cleavages were allowed. The minimum peptide length was defined as seven amino acids. Following modifications were considered: cysteine carbamidomethylation as fixed modification and methionine oxidation and N-terminal acetylation as variable modification. A maximum of five possible modifications per peptide were allowed. The proteolytic peptide filter was applied. Data files generated from the described workflow are hosted in the public repository PRIDE under the identifier PXD037696.

First, efforts were driven to identify global differences in the proteome of neurons located in the dorsal tier and in the ventral tier of the *substantia nigra*, which could explain the selective neuronal vulnerability. For this, the DIA mass spectrometric data of each neuronal population was individual analysed within the software Spectronaut^®^ Pulsar and the resulting numbers of peptides and corresponding proteins were exported. Using this data, the total proteome (proteins identified in at least 1 of the 5 samples) as well as the core proteome (proteins identified in all 5 samples) of each neuronal population were identified.

The obtained core proteomes of neurons located in the dorsal and the ventral tier of the *substantia nigra* were further analyzed in terms of their molecular function and the percentage of a gene ontology (GO) term was calculated and compared between both core proteomes. Therefore, a GO analysis was performed using the PANTHER Classification System of Geneontology Unifying Biology. An enrichment analysis of each core proteome was also performed using the PANTHER Classification System. The enrichment analysis was performed as ‘Panther GO-Slim Molecular Function’ analysis with all *homo sapiens* genes in the dataset as reference list. The test type was the Fisher’s Exact and Bonferroni correction for multiple testing was performed. Results of GO analyses are displayed graphically and in tabular format.

To identify proteins that are specific for one neuronal population (so called black/white proteins), proteins implemented in the core proteome of one group were compared with the protein list of all proteins, which were identified in at least one sample of the other group.

For the differential proteome analysis of dorsal and ventral neurons, extracted abundance values for proteins obtained by the software Spectronaut^TM^ Pulsar were further statistically analysed. First, a quantile normalization was carried out as described by Bolstad et al. [19]. In order to perform this, protein abundances were converted into log(2) values. To determine p-values a paired two-sided t-test was performed. The resulting p-values were adjusted according to Benjamini-Hochberg to determine the Qvalue [20]. Proteins with a Qvalue ≤0.05 and an absolute log2 foldchange ≥ 0.5 were significantly different expressed between both neuronal populations. The results of differentiated proteins were visualized by volcano plots (log2 fold change vs -log10 Qvalue) and box plots (based on quantities).

Additionally, as the *substantia nigra* is the brain region that is mainly affected during PD, possible differential expression levels of proteins associated with PD within the two neuronal populations were investigated. For this, data of proteins included in the ‘Parkison’s disease’ pathway of the KEGG pathway database were reviewed and box plots (based on quantities) were generated for such PD proteins identified in this study.

## Results

In neuropathological studies, a more severe neuronal loss during PD was observed in the ventral tier of the *substantia nigra* than in the dorsal tier. Even if this SNV was already proposed several decades ago, reasons for it are still an open issue. This lack of knowledge is due to the fact that it was not possible to isolate specific neuronal populations out of complex brain tissue in the past. This hurdle was overcome by an LMD based specific isolations of neuronal cell bodies located in the ventral and dorsal tier of the *substantia nigra*. By this, proteomic differences between the two neuronal populations were identified, which could explain the SNV within the *substantia nigra*.

In the study, ventral and dorsal neurons were isolated from a total of 5 human subjects and the cell-specific proteome was analyzed by DIA based mass spectrometry in a donor-specific manner. A whole *substantia nigra* was used to generate a tissue-specific spectral library for data analysis. The median age of the human subjects was 73.4 years (range 57 to 84 years) and 40% of the group were female. The characteristics of the study group are listed in Table 1.

**Fig 2:**
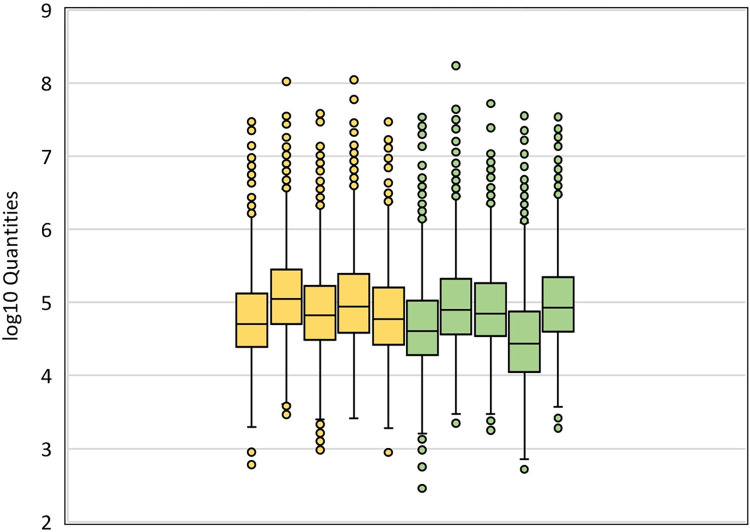
Log10 transformed quantities of each sample. Ventral samples data are marked in yellow and dorsal samples data in green.

Box plots of log10 transformed quantities of all proteins identified in each analyzed ventral and dorsal sample are shown in Fig 2. The individual mass spectrometric datasets of ventral and dorsal neurons were comparable between each individual sample (cell and donor specific) without any significant differences. Thus, the data obtained were of high quality and could be used for the *substantia nigra* neuronal selectivity study.

### Proteome of neurons located in the dorsal tier and in the ventral tier of the *substantia nigra*

A whole *substantia nigra* was used to generate a tissue-specific spectral library for data analysis. The generation of the *substantia nigra*-specific spectral library was described in detail before [21]. In total 69,555 peptides were identified, which could be assigned to 5,391 proteins.

**Fig 3:**
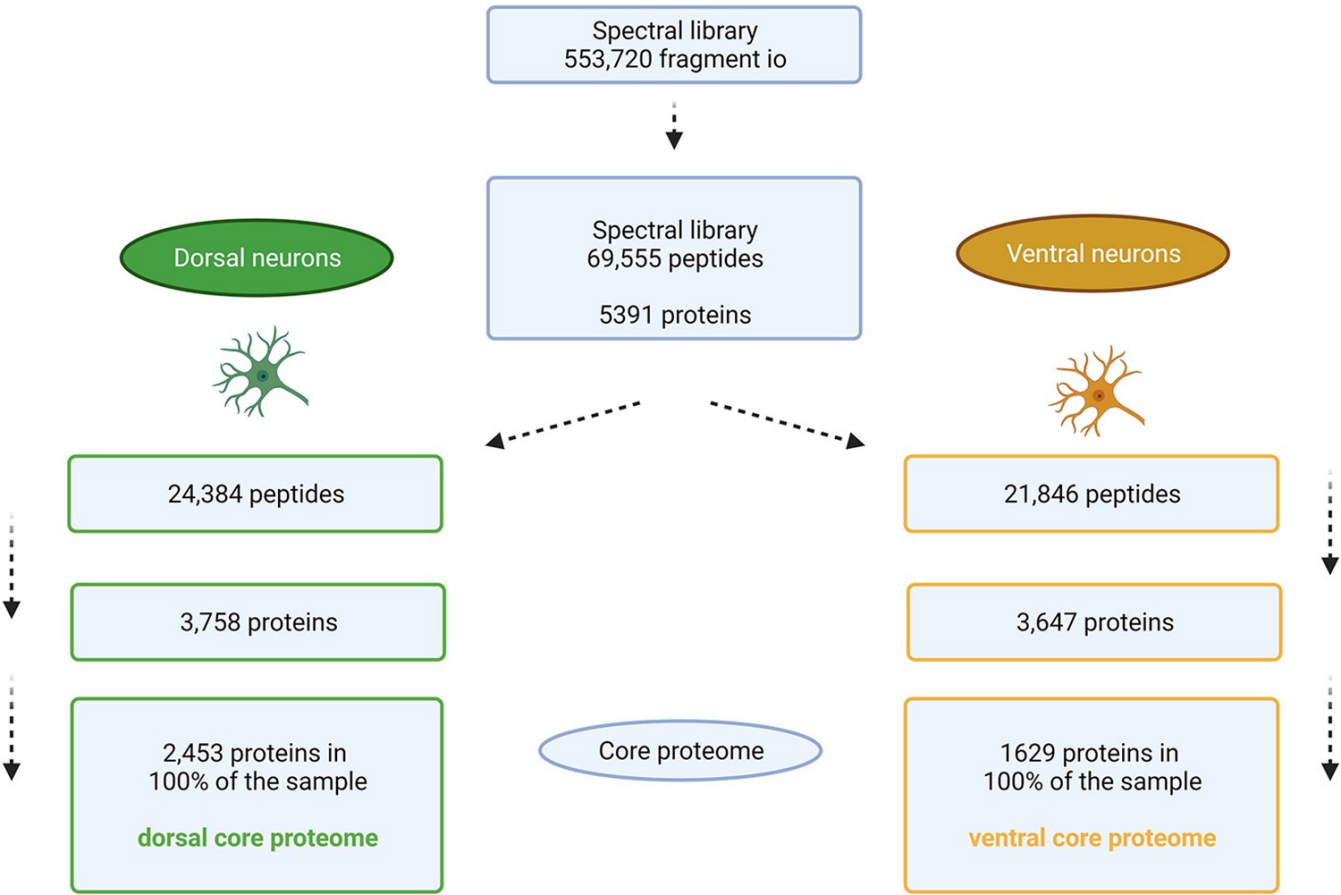
Numbers of proteins identified in the dorsal and ventral neuronal subpopulation of the *substantia nigra*. Visualized are the numbers of quantified peptides, proteins as well as proteins that are present in all samples of one study group. These proteins were defined as the core proteome of the respective group. Created with BioRender.com.

Due to the SNV within the *substantia nigra*, it can be assumed that the proteome of neurons located in the dorsal tier differs from the proteome of neurons in the ventral tier. To identify these differences, the general proteome of each neuronal subpopulation was determined. The results are summarized in Fig 3.

The mass spectrometric analysis of neurons located in the dorsal tier of the *substantia nigra* lead to the quantification of 24,384 peptides, corresponding to 3,758 proteins. From these, 2,453 proteins were present in 100% of the samples of the neuronal subpopulation ‘dorsal’. These proteins were defined as the core proteome of neurons located in the *substantia nigral* dorsal tier.

For neurons located in the ventral tier of the *substantia nigra* following results were obtained: 21,846 peptides corresponding to 3,647 proteins. From these, 1,629 proteins were present in 100% of the samples of the neuronal subpopulation ‘ventral’. These proteins were defined as the core proteome of neurons located in the *substantia nigral* ventral tier. Comparing the results of both neuronal subpopulations revealed 50.58% more proteins in the core proteome of the ‘dorsal’ than in the ‘ventral’ subpopulation.

**Fig 4:**
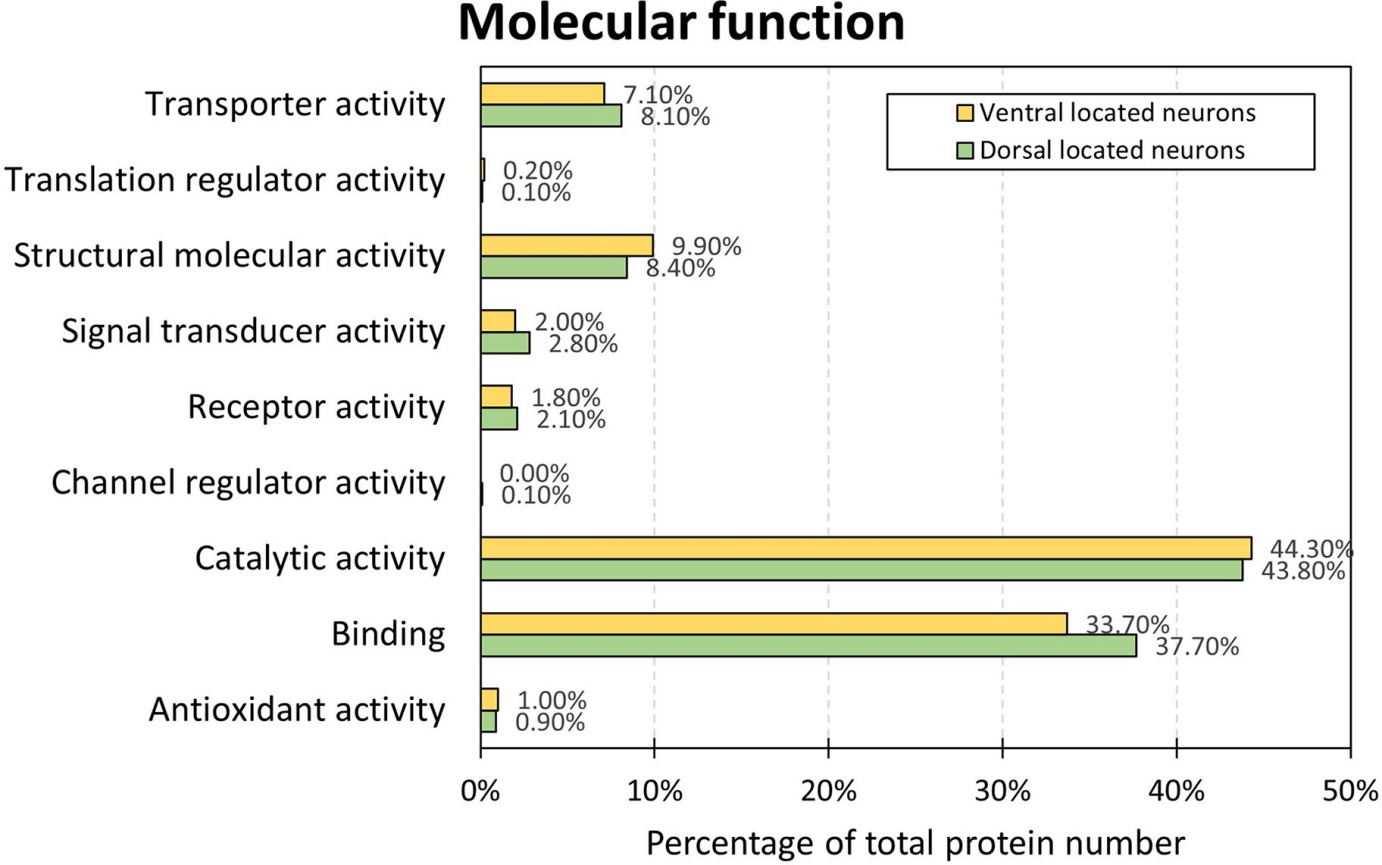
Molecular function of proteins of neurons located in the dorsal and in the ventral tier of the *substantia nigra*. The percentage of proteins associated with the different GO terms concerning their molecular function is visualized. Proteins of neurons in the dorsal tier of the *substantia nigra* are marked in green and proteins of neurons, which are located in the ventral tier of the *substantia nigra*, are marked in yellow.

The results of the descriptive statistical GO analysis for each neuronal subpopulation are given in Fig 4. In both neuronal subgroups the biggest percentage of proteins was associated with the GO term ‘catalytic activity’ and ‘binding’. The only mentionable difference between the two core proteomes was observed for the GO term ‘binding’. The percentage for this GO term was 4% higher for the neuronal subpopulation ‘dorsal’ (37.70%) than for the neuronal subpopulation ‘ventral’ (33.70%).

Also, on molecular function sublevels no other mentionable differences between both neuronal proteomes were observed in the GO enrichment analysis (see Table 3). In the ventral core proteome and in the dorsal core proteome, highest fold enrichments were observed for ‘structural constituent of ribosome’ (5.63 and 4.41, respectively), ‘unfold protein binding’ (4.44 and 3.67, respectively), and ‘structural molecule activity’ (4.44 and 3.53, respectively).

**Table 3:** GO enrichment analysis results.

	*Homo sapiens* (Reference)	Ventral neurons					Dorsal neurons				
		(N = 1,629)					(N = 2,453)				
Molecular Function	Number of proteins	Number of identified proteins	Number of expected proteins	Fold Enrichment	+/-	p-value	Number of identified proteins	Number of expected proteins	Fold Enrichment	+/-	p-value
structural constituent of ribosome	111	49	8.7	5.63	+	6.18E-16	58	13.14	4.41	+	5.02E-14
unfolded protein binding	69	24	5.41	4.44	+	1.63E-05	30	8.17	3.67	+	3.13E-05
structural molecule activity	230	80	18.02	4.44	+	1.91E-21	96	27.22	3.53	+	1.42E-18
ligase activity	119	30	9.32	3.22	+	1.42E-04	54	14.09	3.83	+	5.13E-11
cytoskeletal protein binding	411	94	32.2	2.92	+	8.76E-15	130	48.65	2.67	+	7.03E-17
oxidoreductase activity	431	95	33.77	2.81	+	4.73E-14	110	51.01	2.16	+	7.12E-09
molecular adaptor activity	141	28	11.05	2.53	+	1.85E-02	43	16.69	2.58	+	3.63E-04
small molecule binding	440	84	34.47	2.44	+	2.75E-09	115	52.08	2.21	+	6.12E-10
guanyl nucleotide binding	208	38	16.3	2.33	+	4.94E-03	55	24.62	2.23	+	4.10E-04
tubulin binding	177	32	13.87	2.31	+	3.48E-02	47	20.95	2.24	+	2.52E-03
carbohydrate derivative binding	345	61	27.03	2.26	+	3.08E-05	81	40.84	1.98	+	8.50E-05
cation binding	325	54	25.46	2.12	+	9.55E-04	78	38.47	2.03	+	6.14E-05
ion binding	741	122	58.05	2.1	+	4.75E-10	179	87.71	2.04	+	1.80E-13
RNA binding	617	85	48.34	1.76	+	1.92E-03	117	73.03	1.6	+	2.93E-03
hydrolase activity	1,739	232	136.24	1.7	+	2.88E-11	340	205.84	1.65	+	1.78E-14
catalytic activity	3,916	501	306.79	1.63	+	8.69E-26	731	463.51	1.58	+	1.20E-31
binding	5,893	547	461.67	1.18	+	4.37E-03	793	697.52	1.14	+	3.34E-02
unclassified	10,980	647	860.2	0.75	-	0.00E+00	1,029	1299.64	0.79	-	0.00E+00
signaling receptor regulator activity	276	5	21.62	0.23	-	3.25E-02	5	32.67	0.15	-	6.71E-06
molecular transducer activity	1,085	14	85	0.16	-	3.23E-18	24	128.43	0.19	-	2.06E-25
DNA binding	1,361	17	106.62	0.16	-	5.86E-24	25	161.09	0.16	-	1.52E-36
transcription regulator activity	1,265	10	99.1	0.1	-	4.72E-27	13	149.73	0.09	-	7.59E-43

Because no major difference was observed by a global proteomic comparison, specific or differentially abundant proteins between both neuronal subpopulations were identified and statistically evaluated.

### Differential proteomic analysis between neurons located in the dorsal and in the ventral tier of the *substantia nigra*

To identify proteins, which were specific identified in one neuronal subpopulation, the core proteome of one neuronal subpopulation was compared to the quantified proteins (a protein had to be present in just one sample of the neuronal subpopulation) of the other one (see Table 4). This strict filter criterion was set to ensure that specifically identified proteins are fundamental part of the corresponding neuronal subpopulation proteome. Nevertheless, the raw data is available, along with an Excel spreadsheet containing the data analysis from this manuscript for further examination. Five proteins were identified to be specific for the neuronal subpopulation ‘dorsal’: Immunoglobulin heavy constant gamma 1 (P01857), Tubulin gamma-1 chain (P23258), ADP-ribosylation factor 3 (P61204), Ras-related protein Rab-11A (P62491) and Aldo-keto reductase family 1 member C1 (Q04828). Four proteins were identified to be specific for the neuronal subpopulation ‘ventral’: Immunoglobulin gamma-1 heavy chain (P0DOX5), Calmodulin-like protein 3 (P27482), Alpha cardiac muscle 1 actin (P68032) and ADP-ribosylation factor 1 (P84077).

**Table 4:** Specific proteins for dorsal / ventral located neurons.

Protein name	Protein Uniprot ID	Dorsal neurons	Ventral neurons
**Immunoglobulin heavy constant gamma 1**	P01857	+	**-**
**Immunoglobulin gamma-1 heavy chain**	P0DOX5	**-**	+
**Tubulin gamma-1 chain**	P23258	+	**-**
**Calmodulin-like protein 3**	P27482	**-**	+
**ADP-ribosylation factor 3**	P61204	+	**-**
**Ras-related protein Rab-11A**	P62491	+	**-**
**Actin, alpha cardiac muscle 1**	P68032	**-**	+
**ADP-ribosylation factor 1**	P84077	**-**	+
**Aldo-keto reductase family 1 member C1**	Q04828	+	**-**

**Fig 5:**
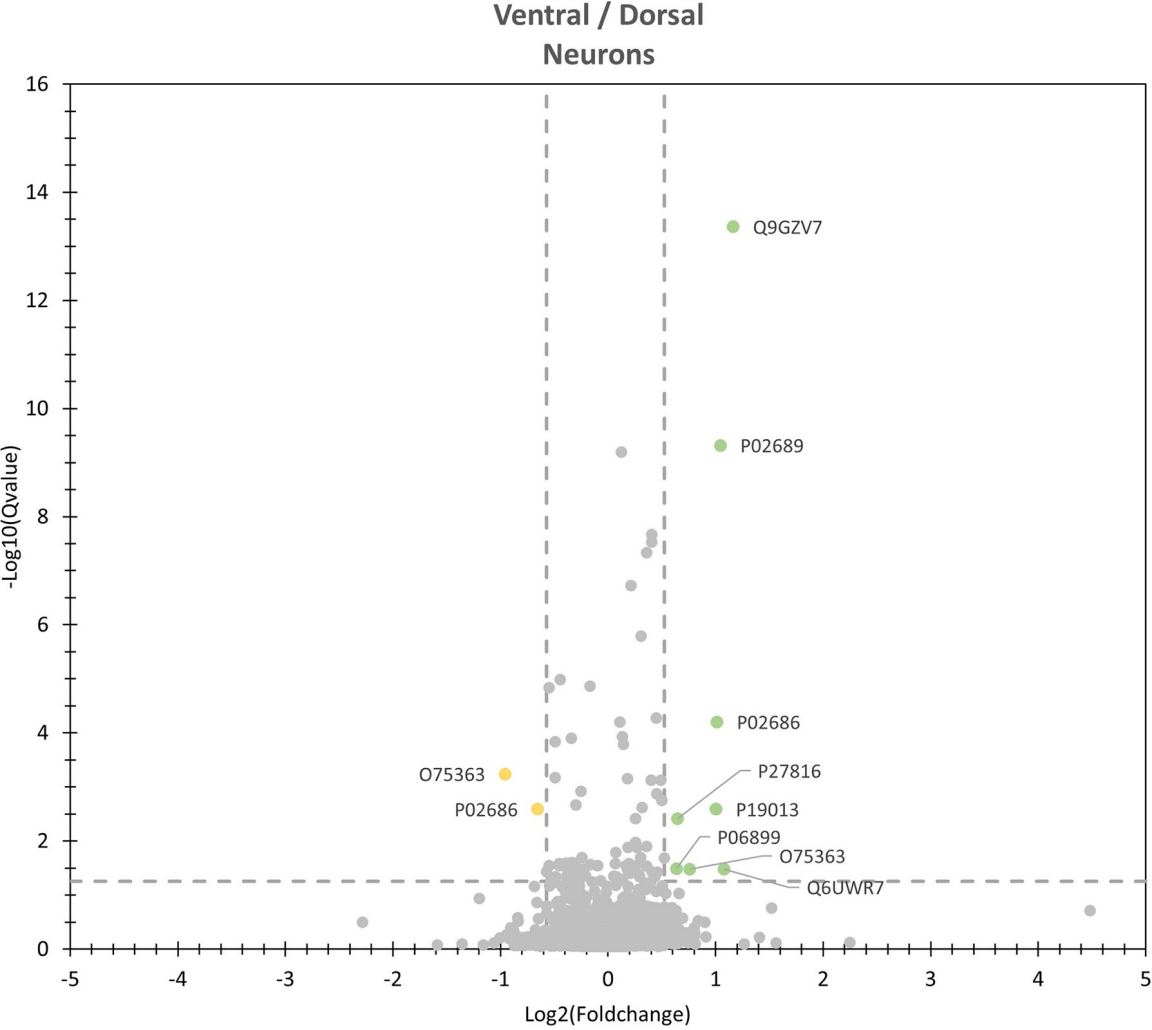
Volcano plot of differentially expressed proteins. Significant differentially expressed proteins are marked in yellow if their abundance is significant higher in ventral neurons and in green if their abundance is significant higher in dorsal neurons. To be classified as significant differentially expressed, proteins had to have a log2 value ≥0.5 and a Qvalue ≤0.05.

**Fig 6:**
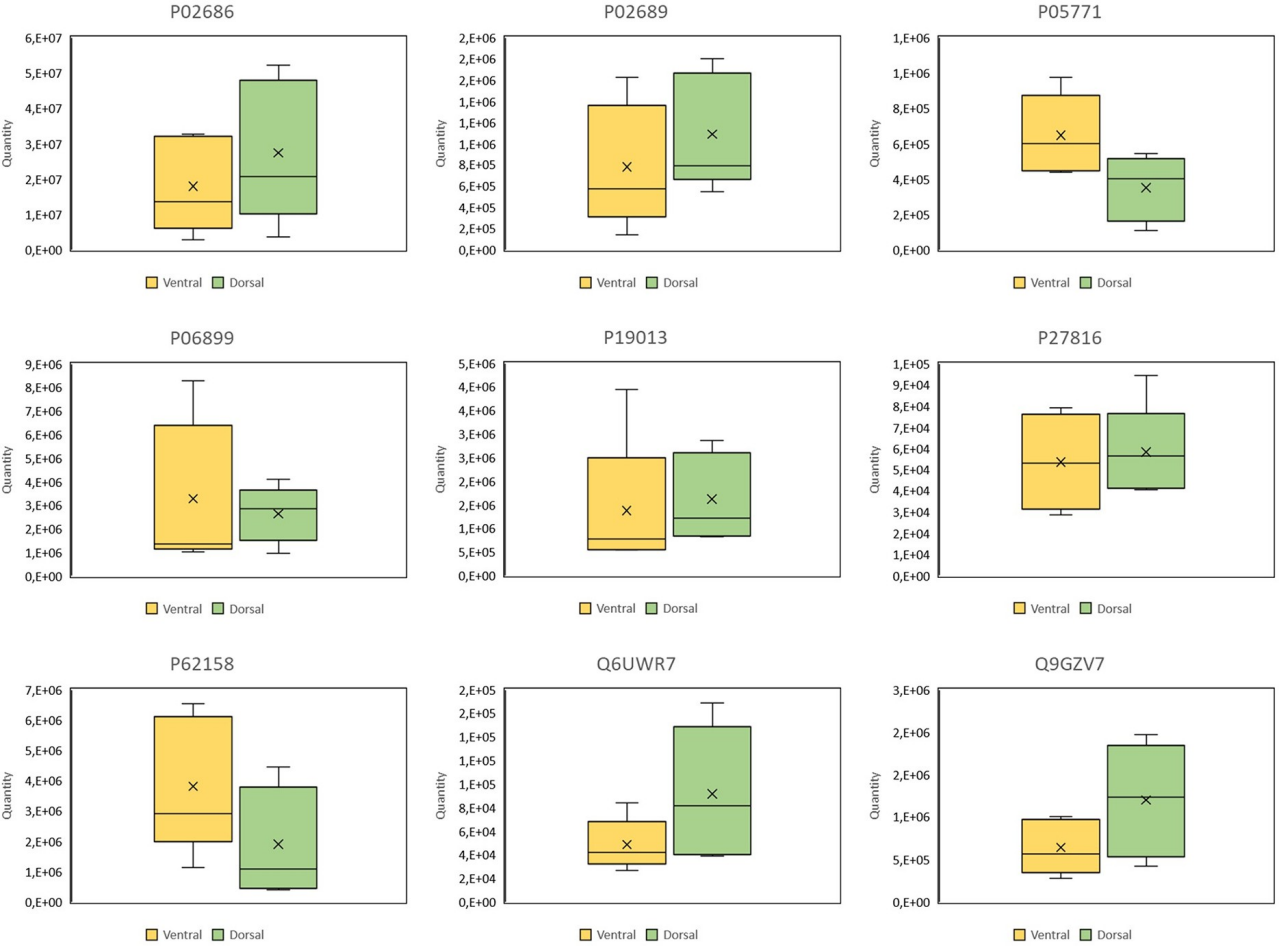
Box plots of differentially expressed proteins. To be classified as significant differentially expressed, proteins had to have a log2 value ≥0.5 and a Qvalue ≤0.05. Protein IDs: P02686 = Myelin basic protein; P02689 = Mylein P2 protein; P05771 = Protein kinase C beta type; P06899: Histone H2B type 1-J; P19013 = Keratin, type II cytoskeletal 4; P27816 = Microtubule-associated protein 4; P62158 = Calmodulin; Q6UWR7 = Ectonucleotide pyrophosphatase/ phosphodiesterase family member 6; Q9GZV7 = Hyaluronan and proteoglycan link protein 2.

The results of the quantitative analysis that was performed to identify differentially expressed proteins are illustrated in Figs 5 and 6 and listed together with the corresponding Qvalues and AVGlog2 ratios in Table 5. Nine differentially expressed proteins were observed. Among them were seven higher expressed in the neuronal subpopulation ‘dorsal’. These proteins were: Myelin basic protein (P02676), Myelin P2 protein (P02689), Histone H2B type 1-J (P06899), Type II cytoskeletal 4 keratin (P19013), Microtubule-associated protein 4 (P27816), Ectonucleotide pyrophosphatase / phosphodiesterase family member 6 (Q6UWR7) and Hyaluronan and proteoglycan link protein 2 (Q9GZV7). Two proteins were higher expressed in the neuronal subpopulation ‘ventral’: Calmodulin (P62158) and Protein kinase C beta type (P05771).

**Table 5:** Differentially expressed proteins.

Protein name	Protein Uniprot ID	Qvalue	Log2 Foldchange	Enriched in
**Myelin basic protein**	P02686	6.30E-05	1.01	Dorsal
**Mylein P2 protein**	P02689	4.81E-10	1.08	Dorsal
**Protein kinase C beta type**	P05771	2.59E-03	0.65	Ventral
**Histone H2B type 1-J**	P06899	3.26E-02	0.64	Dorsal
**Keratin, type II cytoskeletal 4**	P19013	2.59E-03	1.00	Dorsal
**Microtubule-associated protein 4**	P27816	3.38E-03	0.65	Dorsal
**Calmodulin**	P62158	5.77E-04	0.96	Ventral
**Ectonucleotide pyrophosphatase/phosphodiesterase family member 6**	Q6UWR7	3.30E-02	1.05	Dorsal
**Hyaluronan and proteoglycan link protein 2**	Q9GZV7	4.36E-14	1.16	Dorsal

Among the proteins that were higher expressed in the neuronal subpopulation ‘dorsal’ three were associated with the cytoskeleton and neuronal plasticity: Myelin basic protein (P02686), Cytoskeletal type II keratin 4 (P19013), and Microtubule-associated protein 4 (P62158). Also the Aldo-keto reductase family 1 member C1 (Q04828) that was specifically expressed in the same neuronal subpopulation was associated with neuronal plasticity.

Three proteins that were specifically or higher expressed in the neuronal subpopulation ‘ventral’ are associated with calcium. Higher expressed were the proteins Calmodulin (P62158) and Protein kinase C beta type (P05771) and specifically expressed Calmodulin-like protein 3 (P27482).

**Fig 7:**
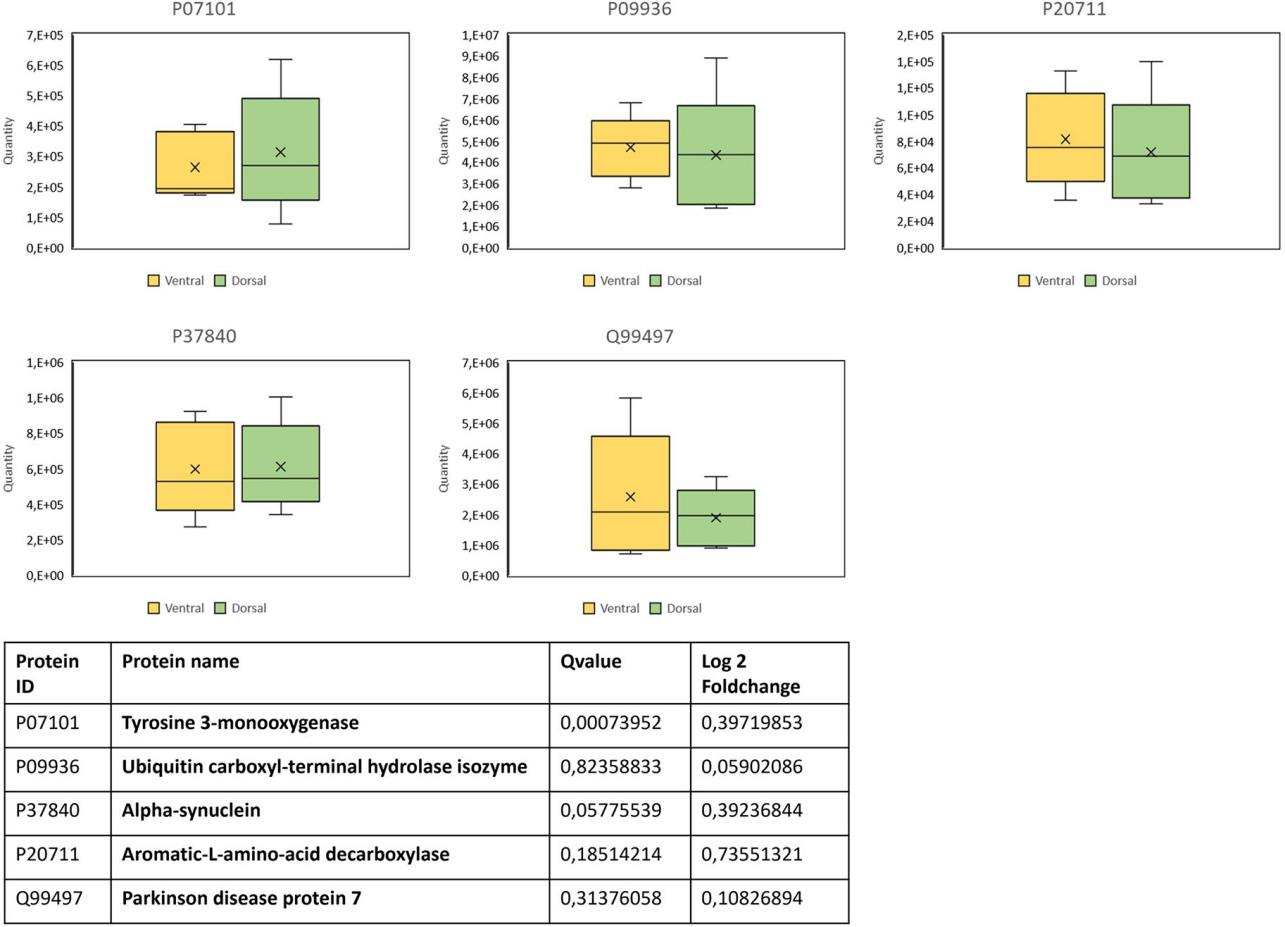
Box plots and data of PD associated proteins according to the KEGG pathway ‘Parkinson’s disease’. To be classified as significant differentially expressed, proteins had to have a log2 value ≥0.5 and a Qvalue ≤0.05. Protein IDs: P07101 = Tyrosine 3-monooxygenase; P09936 = Ubiquitin carboxyl-terminal hydrolase isozyme; P37840 = Alpha-synuclein; P20711 = Aromatic-L-amino-acid decarboxylase; Q99497 = Parkison disease protein 7.

Of 19 proteins that are listed in the KEGG pathway ‘Parkison’s disease’, 5 were identified in this study: Tyrosine 3-monooxygenase (P07101), Ubiquitin carboxyl-terminal hydrolase isozyme (P09936), Alpha-synuclein (P37840), Aromatic L-amino-acid decarboxylase (P20711), and Parkinson disease protein 7 (Q99497). None of these proteins were specifically (so called black/white proteins) or significantly differential expressed between the neurons of the dorsal and the ventral tier of the *substantia nigra* (see Fig 7). Only the Aromatic-L-amino-acid decarboxylase (P20711) had a log 2 foldchange higher than 0.5 (0.74, higher expressed in neurons of the ventral tier of the *substantia nigra*) but its Qvalue was 0.18 thus not fulfilling the filter criteria.

## Discussion

Prior work has documented that SNV refer not only to specific brain regions, which are primarily affected in neurodegenerative diseases, but also to different populations of neurons located in one brain region [3]. Hassler, for example, reported already in 1938 a more severe neuronal loss within the ventral tier of the *substantia nigra* during PD compared to the dorsal tier of the *substantia nigra* [22]. His observations were confirmed by Fearnley *et al*. and Gibb *et al*. [9, 11]. However, these studies were microscopically observations and reasons leading to the SNV are still not fully understood, even though this SNV is known for decades. It is assumed that molecular processes affecting the synaptic function as well as the maintenance of the cytoskeleton play a role in SNV. This is assumed, since especially neurons with longer axons reveal a higher proneness to neurodegeneration [3]. Further, it is predicted that specific proteins have a neuroprotective effect on neurons [3]. For example, Yamada and coworkers as well as Damier *et al*. predict a neuroprotective role of Calbindin D28K in PD [23, 24].

### Dorsal and ventral neuronal proteome

In this study, a proteomic approach based on LMD and DIA mass spectrometry was performed to define the proteome of ventral and dorsal neurons of the *substantia nigra* and based on this to identify proteins, which are either specific or differentially expressed in neurons located in the dorsal or the ventral tier of the *substantia nigra*. Using this approach, it was possible to identify the specific proteome of neurons located in the dorsal and in the ventral tier of the *substantia nigra*. The core proteome of dorsally located neurons includes 2,453 proteins and the core proteome of ventrally located neurons 1,629 proteins. These numbers indicate a higher complexity of the identified core proteome of the neuronal subpopulation ‘dorsal’ which could have two reasons: either a higher adaptivity of dorsally located neurons in *substantia nigra* or a more diverse neuronal population within the ventral subpopulation. Interestingly, focusing on the molecular function of proteins, the overall composition is similar. For both neuronal proteomes, highest fold enrichments were found for the GO terms ‘structural constituent of ribosome’, ‘unfold protein binding’, and ‘structural molecule activity’ (4.44 and 3.53, respectively). This was expected. The neuronal proteome has to be highly adaptive due to the morphological complexity of neuronal tissue and the dynamic network.

Using these cell specific proteomes, proteomic differences between dorsal and ventral neuronal subpopulations were identified, which were masked by the highly complex proteome of the heterogeneous tissue of the whole *substantia nigra*. These proteins may play an essential role in the functionality of neurons and thus their SNV. Further, in the present study, molecular processes, which may cause the SNV within the *substantia nigra* were identified. Five proteins were specific for neurons located in the dorsal tier of the *substantia nigra*: Immunoglobulin heavy constant gamma 1 (P01857), Tubulin gamma-1 chain (P23258), APD-ribosylation factor 3 (P61204), Ras-related protein Rab-11A (P62491) and Aldo-keto reductase family 1 member C1 (Q04828). Additionally, seven proteins were higher expressed in neurons of the dorsal tier: Myelin basic protein (P02686), Myelin P2 protein (P02689), Histone H2B type 1-J (P06899), Keratin type II cytoskeletal 4 (P19013), Microtubule-associated protein 4 (P27816) and Hyaluronan and proteoglycan link protein 2 (Q9GZV7). For neurons of the ventral tier of the *substantia nigra*, four proteins were specific: Immunoglobulin heavy constant gamma 1 (P0DOX5), Calmodulin-like protein 3 (P27482), Actin alpha cardiac muscle 1 (P68032) and ADP-ribosylation factor 1 (P84077). Further, proteins, which were higher expressed in neurons located in the ventral tier were: Calmodulin (P62158) and Protein kinase C beta type (P05771). The possible role of these proteins on the SNV expressed in the *substantia nigra* will be discussed in the following.

None of the PD related proteins that were identified in this study were differentially expressed in this study.

### Specific proteins may maintain the stability of the cytoskeleton and thus the neuronal plasticity of dorsally located *substantia nigral* neurons during PD

Neuronal plasticity is an essential functionality of neurons. It allows neurons to adapt to changing conditions, as they occur during ageing and disease, and by that maintaining their function [25]. Neuronal plasticity is based on the fact that the cytoskeleton of neurons is an adaptive, dynamic and highly complex machinery of proteins and protein complexes rather than one fixed structure [26]. Generally, it can be differentiated between three distinct structural complexes that interact with each other and form the cytoskeleton: microtubules, neurofilaments and microfilaments [27]. Microtubules are a dynamic structural element of the cytoskeleton, which are also involved in the transport of membrane-bounded organelles. Neurofilaments and microfilaments support the neuronal morphology [27]. These three structures interact with each other and additional proteins. By that they execute three general functions of the cytoskeleton: 1. spatially organization of the content of the cell; 2. biochemically and physically connection of the cell to its environment; 3. enabling the movement and structural changes of the cell [28]. These processes maintain the morphology of neurons as well as the signal transmission of neurons [29, 30]. Any dysfunction of this complex system can lead to an altered neuronal function, as e.g. a hindered signal transduction [31]. Since different neurons are exposed to different stimuli, it can be assumed that the cytoskeleton of different neuronal populations varies, too. This leads to the hypothesis that cytoskeletal mechanisms could have an important impact on the SNV. In the context of the SNV within the *substantia nigra* this would mean that neurons in the dorsal tier of the *substantia nigra* may have a higher number of cytoskeletal supportive proteins.

This hypothesis is strengthened by the finding of this study. Three of six proteins, which were higher expressed in neurons located in dorsal tier of the *substantia nigra* compared to those of the ventral tier, are associated with the cytoskeleton and neuronal plasticity: Myelin basic protein (P02686), Cytoskeletal type II keratin 4 (P19013), and Microtubule-associated protein 4 (P27816). Furthermore, one protein that is only expressed in the dorsal neuronal subpopulation is assumed to play a role in neuronal plasticity: Aldo-keto reductase family 1 member C1 (Q04828).

Myelin basic proteins are one of the most abundant proteins of the myelin membrane in the central nervous system [32]. It is known that this protein group forms the myelin sheath and maintains its stability and integrity [33, 34]. Studies revealed that these protein types bind microtubules and cross-link them with actin [33]. This may stabilize the cytoskeleton. Further, the formation of myelin is supported by Aldo-keto reductase family 1 member C1 (Q04828), a protein which is not expressed in neurons that are located in the ventral tier of the *substantia nigra*. Microtubules are also formed, stabilized and cross-linked with actins by the Microtubule-associated protein 4 (P27816) [35], a protein that was identified in this study to be higher expressed in neurons of the dorsal tier. A further finding strengthening the hypothesis that neurons of the dorsal tier of the *substantia nigra* may have a higher number of proteins associated with the cytoskeleton, is the specifically expression of the protein Tubulin gamma-1 chain (P23258) in neurons of the dorsal tier. This protein is a major component of microtubules. Furthermore, the protein Cytoskeletal type II keratin 4 (P19013) was found to be enriched in neurons of the dorsal tier. It belongs to the intermediate filaments and gives the cytoskeleton mechanical strength [36]. Intermediate filaments are highly abundant in axons of motor neurons, which project axons of around 1 meter, and are essential to support this long and thin structures [36].

Summing these findings up, the cytoskeleton of neurons located in the ventral tier of the *substantia nigra* may be more affected by cellular stress, like it occurs during PD, due to a lower number of cytoskeleton supportive proteins. As a result, the functionality of the cytoskeleton, as for example its stability or the signal transmission, may be disturbed. This in turn may lead to the failure of neurons of the ventral tier to respond successfully to alterations, which results in their breakdown. This hypothesis is further strengthened by the higher complexity of the proteome of neurons located in the dorsal tier of the *substantia nigra* (2,453 proteins) compared to the proteome of neurons located in its ventral tier (1,629). Additionally, the percentage of proteins associated with the GO term ‘binding’ is higher for neurons, which are located in the dorsal tier of the *substantia nigra*. These findings indicate that neurons of the dorsal tier of the *substantia nigra* may refer to a higher number of possible strategies to respond to changing conditions in comparison to neurons in the ventral tier. This may explain, why neurons dorsally located in the *substantia nigra* are less prone to degenerative processes of PD.

### Calcium-activated proteins that interact with alpha-synuclein may cause a higher vulnerability of ventrally located neurons in the *substantia nigra* to PD

The neuronal plasticity is also regulated by the calcium homeostasis since a high number of calcium activated kinases are involved in cytoskeletal processes [37]. Further, it is known that a disturbed calcium homeostasis occurs during ageing. Because of this, it can be assumed that different calcium activated proteins may have an impact on neuronal plasticity and by that could be factors leading to SNV. A number of calcium binding proteins are already in the focus of research. Among them are the calpains, a family of calcium-activated cysteine proteases. Previous research linked them to molecular processes of ageing and neurodegenerative diseases, such as PD [38]. Recently, Kamath *et al*. identified ten neuronal populations within the *substantia nigra*. Six of these clusters preferentially expressed calbindin. Two of these calbindin expressing populations, CALB1_GEM and CALB1_TRHR, were strongly enriched in the dorsal tier [15]. An increased calcium concentration within cells leads to a higher rate of activated calpains, which in turn enhances the proteolytic cleavage rates [39]. Another calcium-binding protein is Calbindin D28K. It is in the focus of research, since it is assumed that it has a neuroprotective effect on neurons located in the dorsal tier of the *substantia nigra* in which it is specifically expressed [12]. Further, examinations of Rcom-H’cheo-Gauthier and coworkers demonstrated the absence of alpha-synuclein aggregates in calbindin D28K positive cells [40]. Based on this information, calcium-binding proteins were investigated in more detail in this study to identify a possible linkage between them and the SNV within the *substantia nigra*. Among 18 proteins that were identified to be differentially or specifically expressed in the two different neuronal populations examined in this study, three are associated with calcium: Calmodulin (P62158), Protein kinase C beta type (P05771) and Calmodulin-like protein 3 (P27482). It is to mention that these proteins were either specifically expressed (Calmodulin-like protein 3) or higher expressed (Calmodulin and Protein kinase C beta type) in neurons of the ventral tier of the *substantia nigra* compared to those of the dorsal tier. Of great interest is the higher expression of calmodulin. Calmodulin is involved in cytoskeletal dynamics as it interacts with alpha-synuclein in a calcium-dependent manner [41]. This interaction activates phosphokinases that phosphorylate proteins in the cytoskeleton [41–43]. The interaction of calmodulin and alpha-synuclein triggers the conformational change of alpha-synuclein into a helical structure. Based on these findings, new assumptions about the molecular mechanisms that may lead to SNV within the *substantia nigra* can be made.

**Fig 8:**
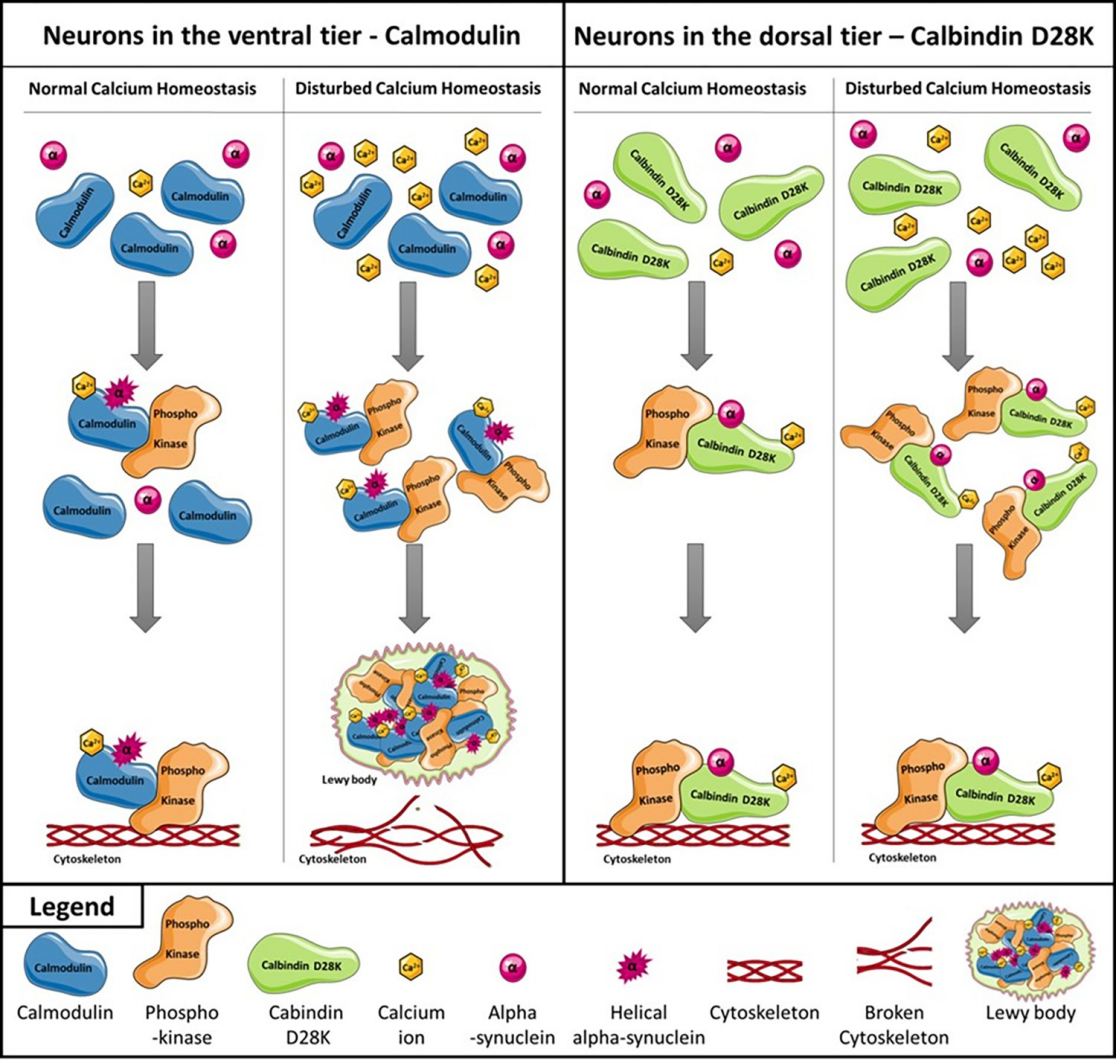
Calcium-dependent interaction of alpha-synuclein with calmodulin and calbindin D28K. Visualized is the interaction of alpha-synuclein with different calcium-dependent proteins. Different interaction partners affect the structure of alpha-synuclein in different ways. While Calmodulin promotes a conformational change of alpha-synuclein, Calbindin D28K prevents alpha-synuclein from this structural change. These differences could be one reason for the SNV between neurons located in the dorsal tier and the ventral tier of the *substantia nigra*.

The calcium-dependent cross-linking of alpha-synuclein and Calmodulin is schematically illustrated in Fig 8. While Calmodulin is activated by the cross-linking and triggers phosphokinases; alpha-synuclein performs a conformational change into a helical structure that may lead to its aggregation [41]. This process may be altered in PD, due to a possible higher concentration of calcium in neurons and/or a mutated form of alpha-synuclein. Regardless of the underlying mechanisms, the formation of Lewy bodies is observed in PD, with alpha-synuclein as the main component [44]. Studies by Iwatsubo and colleagues revealed the presence of a Calmodulin interaction partner (Calmodulin-dependent protein kinase II) in Lewy bodies [44]. This could mean that the whole complex of Calmodulin, alpha-synuclein and an interacting phosphokinase is prone to be included into Lewy bodies. If so, the phosphokinase would be unavailable for molecular processes that maintain the cytoskeletal structure, leading to the destabilization or breakdown of the cytoskeleton. This effect may be strengthened by increased amounts of calcium ions, which would lead to the formation of more calmodulin centered complexes, the incorporation of its involved proteins in Lewy bodies, and by that to a higher vulnerability of neurons located in the dorsal tier of the *substantia nigra*.

In contrast thereto, in neurons of the dorsal tier of the *substantia nigra*, Calbindin D28K is expressed, which plays a similar role than Calmodulin. However, Calbindin D28K inhibits the calcium-dependent conformational change of alpha-synuclein [45]. Thus, it may be that firstly, it inhibits the formation of Lewy bodies and secondly, it ensures the functionality of phosphokinase proteins.

## Outlook

Summarized, only a small number of specific proteins or higher expressed proteins could be identified in this study for neurons located in the dorsal as well as in the ventral tier of the *substantia nigra*. It was noticeable that differentially expressed proteins in neurons located in the dorsal tier of the *substantia nigra* were mainly involved in the cytoskeleton maintenance. Specifically (so called black/white proteins), and differentially expressed proteins of neurons located in the ventral tier of the *substantia nigra*, as Calmodulin, were associated with a disturbed calcium homeostasis and the aggregation of alpha-synuclein. These findings led to a new hypothesis, which indicates that the molecular processes maintaining the cytoskeleton stability may be involved in the SNV within the *substantia nigra*. This hypothesis has to be examined in more detail in the future, especially since it is based on a relatively small cohort study. Thus, to verify the proteomic differences between neuronal populations of the ventral tier and the dorsal tier of the *substantia nigra*, the performed study should be repeated with a bigger cohort. Furthermore, investigations of possible reasons for the SNV of different neuronal populations within the *substantia nigra* revealed that neurons located in the dorsal tier expressed a higher amount of cytoskeleton-associated proteins, which may support the stabilization and maintenance of this network. Due to these findings, the cytoskeleton should be investigated in future studies in more detail. This could be done by the enrichment of the cytoskeletal proteome prior to mass spectrometric analysis. Additional to this, the composition of Lewy bodies and the possible abundance of calmodulin in it, may provide new insights into the calcium-dependent mechanisms which may be involved in the pathomechanisms of PD as well as of the SNV within the *substantia nigra*. The proteomic investigation could be done by an LMD based isolation and enrichment of Lewy bodies.

## Supporting information

S1 Data (XLSX)

## Abbreviations

DIA: Data independent acquisition
FDR: False discovery rate
GO: Gene ontology
HPLC: High performance liquid chromatography
iRT: internal retention time
LMD: Laser microdissection
PD: Parkinson’s disease
SNV: Selective neuronal vulnerability
TFA: Trifluoroacetic acid
